# Culturally adapted psychoeducation for bipolar disorder in a low-resource setting: protocol for a multicentre randomised controlled trial

**DOI:** 10.1192/bjo.2022.598

**Published:** 2022-11-25

**Authors:** M. Ishrat Husain, Madeha Umer, Muqaddas Asif, Ameer B. Khoso, Tayyeba Kiran, Moin Ansari, Huma Aslam, Moti Ram Bhatia, Farasat A. Dogar, M. Omair Husain, Hazrat A. Khan, Ali A. Mufti, Benoit H. Mulsant, Farooq Naeem, Haider A. Naqvi, Claire de Oliviera, M. Sajjad Siddiqui, Asad Tamizuddin, Wei Wang, Juveria Zaheer, Nusrat Husain, Nasim Chaudhry, Imran B. Chaudhry

**Affiliations:** Campbell Family Mental Health Research Institute, Centre for Addiction and Mental Health, Toronto, Ontario, Canada; and Department of Psychiatry, University of Toronto, Toronto, Ontario, Canada; Campbell Family Mental Health Research Institute, Centre for Addiction and Mental Health, Toronto, Ontario, Canada; and Pakistan Institute of Living and Learning, Karachi, Pakistan; Pakistan Institute of Living and Learning, Karachi, Pakistan; Department of Psychiatry, Liaquat University of Medical and Health Sciences, Hyderabad, Pakistan; Department of Psychiatry and Behavioral Sciences, Allama Iqbal Medical College/Jinnah Hospital, Lahore, Pakistan; Department of Psychiatry, Peoples University of Medical and Health Sciences for Women, Nawabshah-Shaheed Benazirabad, Pakistan; Punjab Institute of Mental Health, Lahore, Pakistan; Balochistan Institute of Psychiatry and Behavioral Sciences, Quetta, Pakistan; Jinnah Medical College, Peshawar, Pakistan; Department of Psychiatry, DOW University of Health Sciences, Karachi, Pakistan; National Psychiatric Hospital, Multan, Pakistan; Institute of Psychiatry, WHO Collaborating Centre for Mental Health Research and Training, Rawalpindi, Pakistan; Campbell Family Mental Health Research Institute, Centre for Addiction and Mental Health, Toronto, Ontario, Canada; Division of Psychology and Mental Health, School of Health Sciences, University of Manchester, Manchester, UK; and Mersey Care NHS Foundation Trust, Liverpool, UK; Pakistan Institute of Living and Learning, Karachi, Pakistan; Division of Psychology and Mental Health, School of Health Sciences, University of Manchester, Manchester, UK; and Department of Psychiatry, Ziauddin University, Karachi, Pakistan

**Keywords:** Bipolar disorder, psychoeducation, clinical trial, Pakistan, psychosocial interventions

## Abstract

**Background:**

Bipolar disorder is a source of marked disability, morbidity and premature death. There is a paucity of research on personalised psychosocial interventions for bipolar disorder, especially in low-resource settings. A pilot randomised controlled trial (RCT) of a culturally adapted psychoeducation intervention for bipolar disorder (CaPE) in Pakistan reported higher patient satisfaction, enhanced medication adherence, knowledge and attitudes regarding bipolar disorder, and improvement in mood symptom scores and health-related quality of life measures compared with treatment as usual (TAU).

**Aims:**

The current protocol describes a larger multicentre RCT to confirm the clinical and cost-effectiveness of CaPE in Pakistan. Trial registration: NCT05223959.

**Method:**

A multicentre individual, parallel-arm RCT of CaPE in 300 Pakistani adults with bipolar disorder. Participants over the age of 18, with a diagnosis of bipolar I or II disorder who are currently euthymic, will be recruited from seven sites: Karachi, Lahore, Multan, Rawalpindi, Peshawar, Hyderabad and Quetta. Time to recurrence will be the primary outcome assessed using the Longitudinal Interval Follow-up Evaluation (LIFE). Secondary measures will include mood symptoms, quality of life and functioning, adherence to psychotropic medications, and knowledge and attitudes regarding bipolar disorder.

**Results:**

This trial will assess the effectiveness of the CaPE intervention compared with TAU in reducing the time to recurrence for people with bipolar disorder currently in remission in Pakistan and determine the effect on clinical outcomes, quality of life and functioning.

**Conclusions:**

A successful trial might lead to rapid implementation of CaPE in clinical practice, not only in Pakistan, but also in other low-resource settings, including those in high-income countries, to improve clinical outcomes, social and occupational functioning, and quality of life in South Asian and other minority group patients with bipolar disorder.

Bipolar disorder affects 40 million people worldwide, with a significant disease burden attributed to low- and middle-income countries (LMICs).^1^ It is a highly recurrent condition, with poor recovery between episodes of illness.^2^ Onset is usually in early adult life and over a lifetime bipolar disorder is associated with significant psychosocial impairment and economic costs.^3^ Worldwide, approximately 43% of people with bipolar disorder report suicidal ideation, 21% have a plan for suicide and 16% attempt suicide.^4^ Recurrence rates and hospital admissions for people with bipolar disorder remain high despite advances in psychopharmacology and psychosocial interventions. The Global Burden of Disease studies attributed 9.9 million years lived with disability (YLD) to bipolar disorder between 1990–2010, making it the 16th leading cause of YLD worldwide.^3^ In the USA, the total management and treatment cost for persons with bipolar disorder in 2009 was estimated to be US$151 billion and the cost per patient ranges from US$1904 to $33 090 annually.^5^ Although pharmacotherapy is essential for the successful treatment of bipolar disorder, adjunctive psychosocial interventions are effective for acute depressive episodes, maintenance treatment, relapse prevention and restoration of quality of life to the individual and their family.^6,7^

There is minimal access to psychological and medical treatments for mental health problems in Pakistan.^8^ The psychopathology, incidence, prevalence and course of bipolar disorder are poorly described in LMICs, including Pakistan, although cross-sectional surveys indicate that the prevalence of bipolar spectrum disorders may be as high as 14% in Pakistani youth.^9,10^ Three-quarters of the global burden of mental, neurological and substance use disorders, including bipolar disorder, lies within LMICs, yet 90% of this population do not have access to appropriate mental healthcare^3^ or to mental health research, which is the foundation for building evidence-based, person-centred services. People with mental disorders such as bipolar disorder are far more likely than the general population to die as a consequence of their untreated mental or physical health problems.^11^ There is an urgent need to support research that may enhance the practice of health workers in this setting. There is a need to provide evidence for interventions that can empower patients with knowledge about their mental health in order to achieve and maintain recovery. In Pakistan, the economic burden of mental disorders, including bipolar disorder, was conservatively estimated at over $4 billion in 2006.^12^ This imposes a significant strain on the economy and on severely constrained mental healthcare spending, which in Pakistan is 0.004% of total healthcare spending.^13^ Economic growth and stability in Pakistan are a top priority for the country, but high rates of mental health problems such as bipolar disorder pose a sizeable challenge to this emerging economy.^14^ The World Bank's third edition of disease control priorities includes mental health conditions since effective treatment of these is likely to deliver the greatest economic and social benefit in low-resource settings, reducing disability and enhancing productivity.^15^ There is an urgent need to provide culturally appropriate, evidence-based interventions that can be delivered feasibly in low-resource settings.

## Psychoeducation for bipolar disorder

Evidence suggests that psychoeducation is an effective adjunctive treatment option in bipolar disorder.^16^ Psychoeducation broadly includes providing the patient and family with information about the nature of the illness, its treatments and key coping strategies.^17^ Psychoeducation is an integral part of treatment for psychiatric disorders, which can improve treatment adherence and reduce the risk of recurrence and consequent hospital admission.^18^ Structured psychoeducation programmes include the provision of information about the course and characteristics of the illness (e.g. recurrence rates, trigger factors for recurrence, early recognition of recurrence, symptom management, risk of suicide), treatment (e.g. medication and its side-effects, importance of adherence), lifestyle factors (e.g. stress management, avoidance of substance use) and other key issues (e.g. relevance of pregnancy).^17^ Psychoeducation can be delivered to groups or individuals and it is a pragmatic and cost-effective intervention.^19^ Several systematic reviews have concluded that psychoeducation is an effective form of psychotherapy for bipolar disorder.^20^ A recent network meta-analysis of adjunctive psychotherapy in bipolar disorder showed that psychoeducation was superior to standard care alone in preventing illness recurrence.^21^ Of note, only three small psychoeducation trials included in this meta-analysis were conducted in LMICs.^7,22,23^ Findings from small pilot studies in LMICs indicate that individual psychoeducation may be superior to group psychoeducation in these settings.^7^

We led the first randomised controlled trial (RCT) of a culturally adapted psychoeducation intervention (CaPE) for bipolar disorder in Pakistan.^24^ The results of this pilot RCT showed that CaPE was acceptable and feasible for Pakistani patients with bipolar disorder. Retention in the study was good (79%) and 89% of participants in the CaPE group attended all 12 sessions. Patient satisfaction was higher in the CaPE group relative to TAU (the control condition) (effect size e.s. = 1.41). Further, there were large effect sizes shown for CaPE versus TAU on medication adherence (e.s. = 0.81), knowledge and attitudes regarding bipolar disorder (e.s. = 0.68), mania (e.s. = 1.18), depression (e.s. = 1.17) and quality of life measures (e.s. = 0.88). An adequately powered and longer-duration multicentre RCT is now required to assess the clinical and cost-effectiveness of the CaPE intervention. Building on the successful completion of the pilot RCT, we propose a confirmatory RCT to determine the clinical and cost-effectiveness of CaPE for adults with bipolar disorder, delivered by trained therapists across seven centres in Pakistan. This project has the potential to benefit people with bipolar disorder in the short term by improving mental health. Given the devastating long-term effects of bipolar disorder, and the pivotal stage of life at which it usually manifests, the potential longer-term benefits of the project are enormous. Clinical and cost-effectiveness data will inform the evidence base for scalable interventions for bipolar disorder in low-resource settings not only in Pakistan but also in other countries.

## Study objectives

1. To determine the effectiveness of the CaPE intervention compared with TAU in reducing the time to recurrence for people in Pakistan with bipolar I or II disorder currently in remission.
2. To determine the effect of the CaPE intervention compared with TAU on clinical outcomes, quality of life and functioning.
3. To estimate the cost-effectiveness of CaPE in a low-resource setting (i.e. Pakistan) (if the RCT confirms the effectiveness of CaPE in bipolar disorder).
4. To explore participants’ and therapists’ perspectives on the perceived usefulness of the proposed intervention and to identify the barriers and facilitators of incorporating the intervention into routine clinical practice through process evaluation.

## Method

### Study design and setting

This will be a multicentre, assessor-masked (‘blind’), individual, parallel-arm RCT (trial registration: NCT05223959). Participants will be recruited from psychiatric units of teaching and non-teaching hospitals in seven centres: Karachi (target *n* = 150), Lahore (target *n* = 50), Multan, Rawalpindi, Peshawar, Hyderabad and Quetta (target *n* = 20 at each). By recruiting participants from across the country, we are confident that the sample will be representative of Pakistani patients with bipolar disorder.

### Participants

Participants meeting the eligibility criteria (Appendix 1) will be included in the study.

### The CaPE intervention

CaPE is a manualised intervention for bipolar disorder consisting of 12 one-to-one psychoeducation sessions, one session per week. The decision to deliver the intervention individually is based on our pilot study^24^ and takes into account local cultural norms and expectations regarding the delivery of mental healthcare. From our experience in large trials of psychosocial interventions in Pakistan, participants are uncomfortable sharing experiences of mental illness in group settings because of local beliefs and the stigma associated with mental illness. Each CaPE session (Appendix 2) lasts for approximately 1 h, beginning with a 20–30 min presentation on the topic of the session, followed by a related exercise (e.g. drawing a life chart or compiling a list of potential triggers for recurrence). The manual is a modified and culturally adapted version of the Barcelona Bipolar Disorders Program.^25^ Master's-level clinical psychologists will facilitate all sessions. Pakistan produces over 3000 Master's-level psychologists each year, and these clinicians are an underused resource, making it feasible and economically sustainable for them to deliver CaPE individually. The same psychologist will deliver all 12 sessions to ensure continuity. Psychologists will receive regular weekly supervision from local co-investigators to maintain fidelity.

#### Cultural adaptation of psychoeducation

Our group has culturally adapted interventions for depression, postnatal depression, self-harm and psychosis using mixed methods, in Pakistan and the UK. The framework used considers three areas: assessment and engagement, awareness of cultural factors and adjustments in implementation.^26,27^ Most cultural adaptations of psychological treatments tend to be for implementation rather than content,^28^ and we use the same principle for the CaPE intervention. We use culturally acceptable idioms when explaining the concept, causes and symptoms of bipolar disorder (sessions 1–3), taking into account participants’ lay perceptions of the causes and treatment of mental illness. We also use local folk stories and images (e.g. to explain the concept of multiple perspectives) as well as examples from religious teachings. To improve engagement, we incorporate simple strategies that have worked in the past. These include speaking in the native language (Urdu), using culturally appropriate terms instead of jargon, and establishing a good rapport and a trusting relationship during the sessions. If the participant agrees, we also involve the main carer and family in sessions, particularly sessions 6, 7 and 8 (risks of treatment discontinuation and detection and management of relapse).

### Control condition (TAU)

Local medical, psychiatric and family medicine services provide routine care according to their clinical judgement and available resources. Treatment as usual (TAU) will be ascertained by the participant's treating physician. Research staff will record the nature and intensity of TAU delivered to each participant. In current practice, people with bipolar disorder are not routinely referred for any psychological therapies in Pakistan. TAU for the disorder in Pakistan largely comprises pharmacotherapy.

### Outcome measures

The primary outcome measure is time (in weeks) to recurrence of a mood (depressive/manic/hypomanic/mixed) episode. This will be assessed at each follow-up using the Longitudinal Interval Follow-up Evaluation (LIFE),^29^ an integrated system for assessing the longitudinal course of psychiatric disorders. The assessment will consist of a semi-structured interview, during which an interviewer uses the LIFE to collect detailed psychosocial, psychopathological and treatment information for the follow-up interval. The weekly psychopathology measures are ordinal symptom-based scales with categories defined to match the criteria of DSM-5. Additional secondary measures are listed in Appendix 3.

All scales (both self-report and clinician-rated) will be administered using a tablet, to allow results to be safely and instantly loaded to the study database, with paper versions available if there are technological difficulties. All scales will be translated into Urdu and back-translated into the original language. Most have been used in previous studies in Pakistan, including our pilot.^24^

### Study procedures

We have seven major recruitment centres, each with multiple out-patient psychiatric units. There are five recruitment sites in Karachi, three in Lahore and one each in Multan, Rawalpindi, Peshawar, Hyderabad and Quetta. Psychiatrists will identify potential participants and offer them information about the study. Those who agree will be contacted by the research team, pre-screened and provided detailed information about the study, including procedures protecting privacy and confidentiality. Potential participants will have a chance to ask questions prior to providing informed written consent. Participants will then undergo a screening assessment based on the eligibility criteria outlined in Appendix 1, followed by the baseline assessment. After that, an independent statistician will check the record of baseline measures and will randomise the participant to one of the two arms of the study. The participant will be contacted by the study coordinator at their recruitment centre within 1 week of randomisation to inform them about treatment allocation. The time and date of sessions will be set based on the participant's preference. Participants randomised to the intervention arm will be offered 12 weekly individual sessions. Each session will last for 60 min. All sessions will be delivered by a trained therapist, who will receive 2 months of training from senior therapists before starting the intervention. Assessments will be carried out at baseline and at 3, 6 and 12 months. The assessment scales will be administered in person and/or via secure videoconferencing software (an alternative to in-person assessment to allow for COVID-19 or travel restrictions) by trained research analysts masked to treatment allocation.

#### Randomisation and masking

Participants will be randomised using a block randomisation technique in a 1:1 allocation to CaPE or TAU. Randomisation will be computer generated and use a random permuted block method with variable block sizes. The sample will be stratified by site, bipolar type (I or II) and self-reported biological sex, given the potential differences in recurrence rates in these groups. In line with best practice in RCTs, we have not included further stratification variables.^38^ We will assess other potential prognostic characteristics (age, duration of illness, number of previous episodes, gender) in our *a priori* subgroup analyses.

This will be an assessor-masked RCT. Owing to the nature of the interventions, clinicians in participating centres and the participants themselves cannot be masked to treatment allocation. Before assessments, participants will be asked not to reveal any information about treatment to assessors. To avoid unmasking, outcome assessors will be located separately from treatment providers, and we will assign new outcome assessors in cases of unintentional unmasking. Statistical analysis will be partially masked (knowing treatment groups, but not what each group is). The intention-to-treat analysis plan will help reduce the effect of attrition bias. Finally, registration on Clinicaltrials.gov and publication of results regardless of the outcome will both minimise reporting bias.

### Sample size

From preliminary evidence collected during the pilot RCT,^24^ we estimated the mean time to recurrence as 10 weeks in the TAU group and 45 weeks in the CaPE group. This resulted in a hazard ratio of 4.51. For the purposes of the current study, we assumed a more conservative mean time to recurrence of 21.4 weeks for the TAU group. For a sample size of 240 (120 in each group), we would have more than sufficient power (0.91) to detect a low hazard ratio of 1.60, which approximately corresponds to a mean relapse time of approximately 34.2 weeks in the CaPE group, a difference that would be of clinical importance. The minimum detectable hazard ratio to attain 0.80 power is 1.49. To allow for a possible 20% attrition rate, we aim to recruit 300 participants in total. For within-subgroup analysis, the minimum detectable hazard ratio is 1.67 for larger groups (e.g. males, ~60% of the sample based on pilot-trial data) and 1.88 for smaller groups (e.g. females, ~40% of the sample based on pilot-trial data). For subgroups defined by medium cut-off points, the minimum detectable hazard ratio is 1.75. For the current design, we could only detect between-group differences in treatment effects with adequate power (0.80) when there are drastic group differences, for example a hazard ratio of 1.75 in one group and 3.5 in another. As the effectiveness of CaPE is not yet established, it is premature to further increase our sample size to power subgroup analyses. Nevertheless, preliminary subgroup analysis will inform power calculations for future studies.

### Statistical analysis

The intention-to-treat principle will be used, with participants analysed according to their allocated group. Our primary outcome is time to recurrence observed within 12 months. The treatment effect will be evaluated using the Cox proportional-hazards model with treatment assignment as the primary predictor. Baseline measures of key clinical characteristics (duration of illness, number of previous episodes) and demographics (age, gender, self-reported biological sex) will be controlled in the model. The treatment effect will be illustrated by the estimated hazard ratio to relapse between the two groups. In addition, parametric survival analysis models^38^ will be employed when appropriate. In the widely used Weibull distribution case (for example), the parametric analysis will use a log-linear model on the scale parameter with treatment assignment as the primary predictor while controlling for the set of covariates described above. The regression coefficient on treatment assignment can be interpreted as the log hazard ratio between the two treatment conditions in terms of relapse. This will help estimate the mean time to recurrence for the two groups. As this is a multicentre trial, dependencies for participants from the same centre will be accounted for by including centre as a random effect.^25^ We will investigate levels of missing data, assumptions (e.g. missing at random) and predictors of missingness. Missingness will be addressed using multiple imputations, capitalising on fitting mixed models with maximum likelihood and appropriate sensitivity analyses.^39^ We will also conduct an exploratory mediation analysis to investigate the role of treatment adherence and bipolar disorder type (I or II) on 12-month outcomes. Statistical inference will be conducted using two-tailed tests with 0.05 as the level of significance and 95% confidence intervals with secondary outcomes adjusted for multiple comparisons as necessary. We will use the most appropriate analytic and graphical features of SPSS, STATA and R as relevant.

#### Frequency of analyses

The formal evaluation of the treatment effects will take place after full recruitment and follow-up. During the course of the trial, the trial statistician, masked to treatment allocation, will carry out periodic quality checks of data and a preliminary analysis of efficacy. The interim data analysis will provide additional means for quality assurance of the project without compromising the validity or integrity. The trial will be conducted and reported as per the recommendations of the CONSORT statement for RCTs.

#### Subgroup analyses

Subgroup analysis will be carried out with respect to age, duration of illness, number of previous episodes, gender and self-reported biological sex by adding a treatment with covariate interaction into the primary analysis model.

#### Cost–utility analysis

A cost–utility analysis from healthcare system and societal perspectives will be conducted to establish the cost-effectiveness of CaPE compared with TAU. The treatment groups will be compared with each other at baseline and then at subsequent follow-ups at 3, 6 and 12 months. EuroQol-5D data will be collected at these time points to measure patient quality of life; quality adjusted life-year (QALY) gains between groups will then be estimated and compared at baseline and at the end of the 12-month period. The costs of delivering CaPE and TAU will be collected and will include costs of staff time, including that of therapists and their training to deliver the intervention, resources and equipment used, and any remuneration given to patients for their time. Data on the direct and indirect costs to patients will be collected using a bespoke questionnaire and will include any additional equipment/aids paid out-of-pocket by patients for the sessions as well as time taken off work by the patient and the carer (if any) to attend sessions. Additional data regarding health service use and direct costs to the healthcare system will also be obtained using the Client Service Receipt Inventory, completed by both treatment groups at 3, 6 and 12 months. We will use standard published sources for unit costs where available and supplement this with local sources to convert resource use into monetary values. Costs will be adjusted for inflation to 2025 Pakistani rupees using appropriate inflators. To estimate the cost-effectiveness of CaPE, an incremental cost-effectiveness ratio will be calculated, defined as the difference in costs divided by the difference in QALYs between the treatment groups. Standard procedures will be followed for imputation of missing values as well as for analysis of uncertainty and non-normal distributions. Further statistical analysis of costs will be performed to test for statistical significance of results, and bootstrapping will be performed to estimate variability in key parameters. The robustness of each treatment will be shown through 95% confidence intervals around the cost-effectiveness ratio, with sensitivity analyses.

### Process evaluation

Effectiveness trials are being increasingly complemented by process evaluation, which offers important information to enhance understanding of trial findings. Process evaluation helps to improve understanding of interventions by exploring barriers and facilitators of implementation, perceived mechanisms of effect and context.^40^ Process evaluation will be guided by the Consolidated Framework for Implementation Research (CFIR) to explore:

- perceived usefulness of the CaPe intervention
- barriers and facilitators of incorporating the CaPe intervention into the everyday work of health professionals
- sustainability of the CaPe intervention in routine practice.

In-depth, one-to-one interviews will be conducted with four groups of participants:

- patients from the intervention arm (*n* = 15–20)
- carers (*n* = 15–20)
- all therapists involved in the trial
- mental health professionals (both psychiatrists and psychologists) (*n* = 10–15).

For participants and carers, maximum variation sampling in terms of different socioeconomic backgrounds, ages, education, gender, geographical areas and clinical profiles (recovery and clinical scores) will be ensured to explore a wide range of experiences. Separate topic guides will be developed by the research team for each group of participants using the CFIR approach. Interviews will be digitally recorded, transcribed verbatim and translated into English. Each interview will last for approximately 60 min. Interviews will be analysed using thematic analysis. Data will be coded by the researchers working independently. A system for regular team meetings will be in place to develop a provisional framework for each set of participants that will be further amended through constant comparison of new data. The final coding framework will be presented to the study team, who will comment on ambiguities and ensure that the framework remains grounded in the data. Analysis will occur in parallel to data collection. The whole team will agree when data saturation has been reached.

### Ethical considerations

Full ethics approval has been received from National Bioethics Committee of Pakistan and the Centre for Addiction and Mental Health (CAMH) Research Ethics Board, Toronto, Canada (REB #138/2021). All members of the research team will comply with the International Conference on Harmonization Good Clinical Practice (ICH-GCP) Guideline. Research staff will be trained in good clinical practice (GCP) and will not begin data collection until the GCP certification is successfully completed. All information provided by the participants will be kept confidential and authorisation will be required prior to access. Patient-identifying information will not be published. Participation in the trial will be voluntary and participants will have the right to withdraw from the study at any time, without giving any reason. Withdrawal from the trial will have no effect on routine care. Informed consent forms will be in the local language for those participants who can read. Participants who are unable to read and write will be provided with verbal information (in the local language) that will encompass all aspects of the participants’ written informed consent form. The consent form will then be signed by the participant and a caregiver will sign alongside. If the participant cannot write, their caregiver will sign alongside the participant's thumbprint. The participant information sheet and the participant informed consent form will include details of the purpose of the study; the opportunity to ask questions; voluntary participation in the study and the right to withdraw from the study at any time, for any reason; and privacy and confidentiality. All participants can decide to participate right away if they want to, or they will be given at least 48 h to read the forms and discuss queries with family or the research team before a decision is made.

#### Safety

We do not anticipate any major risks related to the intervention. However, we acknowledge that potentially vulnerable people with mental illness will be participating in the trial. Some topics discussed or questions asked in the sessions/assessments may cause distress, and in such instances, appropriate support will be provided. We will map available mental health resources, ensuring pathways to care. The research team has considerable clinical and research experience, including the management of difficult situations arising in research interventions and interviews. If any participants recruited to this study experience active suicidal ideation or are subsequently identified as needing treatment that is more intensive they will be appropriately referred for psychiatric evaluation and treatment. Participants in the TAU group identified as needing intensive treatment will be referred to appropriate treatment services after trial completion. Research staff and therapists will be provided with regular supervision throughout the duration of the project. We already have study protocols in place that include details about how to manage difficult situations arising in relation to research, safety and lone working arrangements. In the event of any study-related injury or adverse event, intervention and treatment will be available from medical staff. Any adverse events will be reported to the CAMH Research Ethics Board within the required time frame.

### Data management and monitoring

Each participant will be assigned a unique study identification (ID) number and identifying information will be placed in locked cupboards separate from other study data, which will only be accessible to the authorised researchers. Paper copies of assessment tools will all be stored in locked filing cabinets on Pakistan Institute of Living and Learning (PILL) premises. All anonymised data will be stored on encrypted and password-protected computers. We will convene an independently chaired trial steering committee (TSC) to approve and provide oversight of the trial throughout its various stages. The TSC will include the principal investigator, patient representative, local co-investigators, an independent statistician and an independent chair. The patient representative will be a patient volunteer from the Patient Advisory Committee at the local partner organisation, i.e. PILL. The independent statistician and independent chair will be local experts in the design and execution of clinical trials, and will be approached by study investigators. The Data Safety and Monitoring Committee (DSMC) will monitor data and advise the TSC on whether there are any ethical or safety reasons why the trial should not continue. Both committees will meet twice in the first year and then once a year thereafter.

## Discussion

Our own and others’ data from pilot RCTs suggest that psychoeducation in the maintenance phase of bipolar disorder is an effective adjunctive treatment option in low-resource settings, including LMICs.^20,24,41^ We now need to confirm these preliminary findings and extend this pilot work to confirm the effectiveness and cost-effectiveness of CaPE to inform the scalability and sustainability of the intervention in low-resource settings. Psychoeducation as an intervention provides greater insight to the patient and their family/carers, as well as helping to establish a differential understanding of an illness. Lack of information, particularly after diagnosis of a severe mental illness, may render both the patient and their family helpless. A combination of disease-specific (recognition and management of recurrence) and general information (communication skills training, stressor identification and problem-solving skills) is needed to overcome illness-related difficulties. Since psychoeducation is an approach that combines health psychology with psychotherapy, it results in imparting knowledge, while simultaneously equipping the patient and their family with the necessary skills to manage the condition.^42^ Psychoeducation interventions have evolved into sophisticated programmes catering to wide clinical populations, including people with bipolar disorder.^24,43^ Implications of psychoeducation extend beyond maintenance of remission: cost–benefit analyses reveal that as health outcomes improve, healthcare costs are substantially reduced.^44^ Despite these clear benefits, to our knowledge, there are only limited, underpowered studies of psychoeducation from LMICs, including Pakistan. Therefore, the study described here aims to address a scientific research gap on psychosocial interventions for bipolar disorder in low-resource settings such as LMICs. The study could generate convincing data to persuade guideline makers, key opinion leaders, clinicians and patients to include CaPE as a maintenance treatment option for bipolar disorder in Pakistan. It will also enhance information, scientific knowledge, clinical practice and health policy relating to treatment of bipolar disorder in Pakistan and low-resource settings in both low- and high-income countries. Finally, integrating patients and carers into the study strengthens research capabilities in Pakistan by ensuring that future research is oriented towards patient priorities and development of funding bids for collaborative research.

## Data Availability

Data availability is not applicable to this article as no new data were created or analysed in this report.

## Appendix 1

### Eligibility criteria

Inclusion criteria

- Adult out-patients age 18 years and above
- Diagnosis of DSM-5 bipolar disorder (both bipolar disorder I and bipolar disorder II), currently in remission, confirmed by Structured Clinical Interview for DSM-5 (SCID)
- Currently euthymic (17-item Hamilton Rating Scale for Depression score <8 and Young Mania Rating Scale score <8)
- Able to give informed consent
- On stable psychiatric medication regimen for at least 3 months
- Resident of the trial catchment area.

Exclusion criteria

- Active substance use disorder, based on DSM-5 criteria
- Currently experiencing recurrence (mania, hypomania, mixed or depressive
- Active suicidal ideation
- Unstable residential arrangements that reduce likelihood of being available for the duration of trial.

## Appendix 2

### Sessions of the culturally adapted psychoeducation (CaPE) intervention

Session 1: Concept and causes

Session 2: Symptoms (mania, hypomania, depression and mixed conditions)

Session 3: Evolution and prognosis, psychoactive substance misuse

Session 4: Treatment with medication (mood stabilisers, antipsychotics, antidepressants)

Session 5: Alternatives therapies

Session 6: Risks associated with interruption of treatment

Session 7: Learning to detect early symptoms of relapse

Session 8: What to do when a relapse is detected

Session 9: Regularity of habits

Session 10: Stress control

Session 11: Problem-solving strategy

Session 12: Final session

## Appendix 3

### Assessment schedule

**Table:**

Instrument	Baseline	Month 3	Month 6	Month 12
Demographic questionnaire	✓			
SCID Longitudinal Interval Follow-up Evaluation (LIFE)^29^	✓	✓	✓	✓
Young Mania Rating Scale (YMRS)^30^	✓	✓	✓	✓
17-item Hamilton Rating Scale for Depression (HRSD-17)^31^	✓	✓	✓	✓
EuroQol-5D (EQ- 5D)^32^	✓	✓	✓	✓
Bipolar Recovery Questionnaire (BRQ)^33^	✓	✓	✓	✓
4-item Morisky Medication Adherence Scale (MMAS-4)^34^	✓	✓	✓	✓
Bipolar Knowledge and Attitudes Questionnaire (BKAQ)^33^	✓	✓	✓	✓
Client Service Receipt Inventory (CSRI)^35^	✓	✓	✓	✓
Childhood Trauma Questionnaire (CTQ)^36^	✓	✓	✓	✓
Illness Perception Questionnaire-Revised^37^	✓	✓	✓	✓
