# Culturally adapted psychoeducation for bipolar disorder in a low-resource setting: protocol for a multicentre randomised controlled trial – ERRATUM

**DOI:** 10.1192/bjo.2023.16

**Published:** 2023-02-22

**Authors:** M. Ishrat Husain, Madeha Umer, Muqaddas Asif, Ameer B. Khoso, Tayyeba Kiran, Moin Ansari, Huma Aslam, Moti Ram Bhatia, Farasat A. Dogar, M. Omair Husain, Hazrat A. Khan, Ali A. Mufti, Benoit H. Mulsant, Farooq Naeem, Haider A. Naqvi, Claire de Oliveira, M. Sajjad Siddiqui, Asad Tamizuddin, Wei Wang, Juveria Zaheer, Nusrat Husain, Nasim Chaudhry, Imran B. Chaudhry

In the above article, one of the co-author's surnames was spelled incorrectly. Claire de Oliviera should have been printed as Claire de Oliveira. This has now been updated in the original article online.

The publisher apologises for this error.
